# Cognitive Impairment as A Vulnerability for Exploitation: A Scoping Review

**DOI:** 10.1177/15248380241282993

**Published:** 2024-10-01

**Authors:** Imogen Lambert, Nicola Wright, Alison Gardner, Rachel Fyson, Aisha Abubakar, Rachael Clawson

**Affiliations:** 1 University of Nottingham, UK

**Keywords:** sexual abuse, child abuse, cultural contexts, elder abuse, prostitution/sex work, PTSD, sexual assault

## Abstract

Exploitation is a form of abuse that occurs when one person unfairly manipulates another for profit or personal gain. Various individual and social characteristics have the potential to increase an individual’s risk of being exploited. Cognitive impairment is one potential vulnerability factor that has received minimal research attention. This scoping review aimed to investigate cognitive impairment as a factor that may increase an individual’s vulnerability to exploitation. Study inclusion criteria were: (a) empirical studies; (b) studies presenting extractable data related to cognitive impairment and exploitation; (c) studies exploring cognitive impairment as a vulnerability factor for exploitation; (d) studies published after 1998; and (e) studies available in English. A six-step search strategy was employed: (a) electronic searches of bibliographic databases; (b) screening reference lists of included studies; (c) forward citation tracking in Google Scholar; (d) expert recommendations; (e) website searches of relevant Non-Governmental Organizations (NGOs); and (f) a call for evidence. Twenty studies met the inclusion criteria. Three types of exploitation were reported: sexual (*n* = 10), financial (*n* = 8), and criminal (*n* = 2). Intellectual disability (*n* = 8) and mental health (*n* = 8) were the most frequently described forms of cognitive impairment. The results indicate that cognitive impairment is a factor that increases vulnerability to exploitation. However, the limited number and disparate nature of the studies means that it is impossible to disentangle all the complexities in the relationship between cognitive impairment and exploitation. Further research is needed to understand if cognitive impairment increases vulnerability to all types of exploitation or if it results in varying levels of susceptibility to different types of exploitation.

## Introduction

Exploitation is a form of abuse. Exploitation takes place when one person, either opportunistically or premeditatedly, unfairly manipulates another person for profit or personal gain, including financial, social, or political recompense. A victim may be exploited by one individual or group of individuals, and exploitation may take the form of coerced criminal, sexual, financial, spiritual, or labor-related activities. It has been argued that exploitation is often nurtured within social networks and subcultures in which initial relationships of trust and loyalty (or even friendship and love) are exploited, and exploitative behaviors become normalized (Roe-Sepowitz et al., 2014). Some observers increasingly regard exploitation as a continuum, ranging from extreme forms such as slavery, servitude, and forced/compulsory labor—in which the victim loses control over many/most aspects of their life—to less all-encompassing, but by no means insignificant, crimes involving property or finance (Skrivankova, 2010). Research evidence suggests that exploitation of an individual may increase over time, with victims subjected to experiences and conditions that gradually worsen (Boersma & Nolan, 2022). Research also suggests that exploitation normally occurs in the context of a power disparity (UN, 2017; Wake & Reed, 2019), wherein the relative powerlessness of the victim is taken advantage of by the more powerful exploiter. Vulnerabilities that have been identified as having the potential to increase an individual’s risk of being exploited include a wide range of factors such as age, disability, sex/gender, poverty and financial need, and citizenship status.

Some aspects of vulnerability to exploitation are beginning to be relatively well understood. For example, in the United Kingdom, “failed” asylum seekers and irregular migrants who have neither the legal right to work nor any access to public services are known to be at an increased risk of labor exploitation (Latham-Sprinkle et al., 2019; Lewis et al., 2015; Waite, 2017). Similarly, it is increasingly well-established that physically disabled people are at higher risk of exploitative familiarity, including “mate crime” (where victims are exploited by people who pretend to be their friends) and “cuckooing” (also known as “home-based exploitation”, where the victim’s property is taken over, often for illegal purposes such as drug dealing) (Doherty, 2020; Macdonald et al., 2022). An exploratory study of one English local authority in the United Kingdom found that people with intellectual disabilities and/or mental health issues were particularly vulnerable to exploitation: 30% of all cases of exploitation recorded that the victim was known to have either an intellectual disability and/or mental health issues, and in a further 26% of cases, professionals involved in the case believed that the victim had an undiagnosed intellectual disability and/or mental health issues, which had contributed to their vulnerability (Robinson et al., 2021). Connecting and broadening intellectual disability and mental ill-health as aspects of the wider category of “cognitive impairment”, this review is an attempt to scope the current state of research knowledge regarding the relationship between cognitive impairment and vulnerability to exploitation.

For this review, cognitive impairment has been defined as broadly as possible, to include both developmental and acquired impairment affecting one or more of the six domains of cognitive function set out in The Diagnostic and Statistical Manual of Mental Disorders, Fifth Edition (DSM-V) (albeit specifically in relation to dementia), that is, executive function, learning and memory, perceptual-motor function, language, complex attention, and social cognition (American Psychiatric Association, 2013). This meant that conditions as varied as intellectual disability, dementia, brain injury, autistic spectrum disorders, Attention Deficit Hyperactivity Disorder (ADHD), functional mental health disorders, and substance misuse were included in the search. This wide remit aimed to ensure that the intersections between a broad range of conditions and exploitation could be explored, including those which may often be overlooked (Robinson et al., 2021). It is acknowledged that these diverse conditions may affect an individual’s cognition in different ways. For example, profound intellectual disability or late-stage dementia may affect all six domains of cognitive function noted above. By way of contrast, autistic spectrum disorder or neurodivergence may affect fewer domains, often in more subtle ways (McGee, 2012). Functional mental health disorders may have either an ongoing or an intermittent impact on an individual’s capacity and functioning (Castaneda et al., 2011; Vicent-Gil & Portella, 2021). Substance misuse may for some, only affect cognition when an individual is directly under the influence of drugs or alcohol (Bruijnen et al., 2019).

### A Note on Terminology

As this scoping review has been undertaken by United Kingdom-based academics, United Kingdom terminology relating to exploitation has been used throughout, except when directly quoting from work derived from other national contexts. For the benefit of international readers, the term “labor exploitation” is used in the United Kingdom to refer to the phenomenon which in the United States and some other countries is commonly called “forced labor”, and the term “criminal exploitation” is used in the United Kingdom, which refers to what may elsewhere be known as “forced criminality”.

The term “intellectual disability” is used throughout as this term is most widely understood internationally. Additionally, while “learning disability” is more commonly used in the United Kingdom, it has different meanings in other national contexts. (Cluley, 2017).

### Review Aim

To investigate cognitive impairment as a factor that may increase an individual’s vulnerability to exploitation.

### Review Objectives

1. To develop an overview of the range of literature available that has explored cognitive impairment as a possible factor that increases an individual’s vulnerability to exploitation.
2. To identify knowledge gaps and areas for future research.

## Methodology

To address the study’s aim and objectives, a five-stage review process following the Preferred Reporting Items for Systematic Reviews and Meta-Analyses extension for Scoping Reviews was used (Tricco et al., 2018). The scoping review methodology was selected due to the extensive and heterogenous nature of the subject matter. In comparison to systematic reviews, scoping reviews answer broader questions about the size, variety, and nature of the evidence base. Mai et al. (2014) suggest that scoping reviews are widely viewed as useful starting points for synthesizing, overviewing, and summarizing diverse evidence and information in a particular area. To aid transparency in reporting, the protocol for the scoping review was prospectively published on the study website: https://exploitationandci.org/scoping-review/.

### Search Strategy

A multi-stage search strategy was used to identify relevant literature for inclusion. This comprised of the following: (a) an electronic search of 12 bibliographic databases; (b) screening of reference lists of included studies; (c) forward citation tracking in Google Scholar; (d) expert recommendation; and (e) website searches of relevant United Kingdom Non-Governmental Organizations (NGOs). Due to challenges in establishing a representative sample and considering the available resources, NGOs were selected if the focus of their work met the review’s definition of cognitive impairment or exploitation, were based in the United Kingdom, and had a national focus (United Kingdom-wide or devolved nations)—organizations were identified via a rapid web search, expert recommendations, and a call for evidence distributed via social media and project partner organizations—the Human Trafficking Foundation (http://www.humantraffickingfoundation.org) and the Ann Craft Trust (http://www.anncrafttrust.org).

A controlled vocabulary index and free text terms were used to search the electronic databases. This included terms relating to exploitation (e.g., “exp. Exploit*,” “exp. modern slavery,” “exp. human traffick*,” “slav*,” “sexual exploit*,” “forced labo*”), cognitive impairment (e.g., cognitive AND [impair* OR disability* OR declin*]), intellectual disability (e.g., intellectual OR learning AND [impair* OR difficult* OR disabilit*]), and mental health (e.g., “exp. mental health,” “mental disorder*,” “mental illness*,” “schizo*,” “suicid*”). The search terms were developed in consultation with the Library Support Team at the University of Nottingham. Free text terms were used in cases where a controlled vocabulary index did not exist for a database or website.

Searches were limited to retrieving citations post-1998 when the Human Rights Act came into force in the United Kingdom. This cut-off date was chosen due to the importance of the Human Rights Act in both prohibiting slavery and forced labor (Human Rights Act, 1998, Article 14) and providing a legal means of redress for individuals whose human rights have been violated, whether through exploitation or otherwise. Since its inception, the Human Rights Act has remained influential, being used both as the basis of compensation cases for individuals whose rights have been violated and by professionals (e.g., social workers) as part of the framework for assessing whether to intervene when a person with cognitive impairment is identified as having potentially been exploited.

The searches were completed in a two-week period in January 2023. All identified citations were downloaded into a reference managing software Mendeley (Elsevier, London).

### Selection Criteria

Consistent with a scoping review approach, the inclusion criteria were broad and comprised of the following for both peer-reviewed and grey literature (i.e., NGO reports). Papers which met the inclusion criteria were those which:

(1) reported original data collected via quantitative, qualitative, or mixed methodological approaches;
(2) presented extractable data related to people who have a cognitive impairment and have experienced exploitation; and
(3) explored cognitive impairment as a vulnerability or precipitating factor for exploitation.

To be clear, papers did not need to have exploitation and/or cognitive impairment as their sole or main focus, but they did need to contain extractable data relating to these two factors.

While the electronic database searches were global in focus, the language abilities of the research team meant that only papers written in English could be included in the review. Other reasons for exclusion were: (a) published prior to 1998; (b) single case studies, conference abstracts, editorials, opinion articles, book reviews, literature reviews, theses, or dissertations; (c) presented no extractable data related to people with cognitive impairment who have experienced exploitation; (d) presented data related to people with cognitive impairments but there was no evidence of exploitation; (e) presented data related to exploitation but there was no evidence of cognitive impairment; or (f) discussed cognitive impairment only as a consequence of exploitation. Studies that were correlational and did not indicate whether cognitive impairment was a *vulnerability factor for* exploitation or had arisen as a *consequence of* exploitation were also excluded.

### Screening and Data Extraction

After the removal of duplicates, the titles and abstracts of all identified citations were screened by one reviewer. A second reviewer checked 20% or 250 returns (whichever was the lower). Full texts were single-screened, with the first 20% or 20 papers (whichever was the lower) reviewed by a second reviewer. Data extraction was completed using the Qualtrics survey software. The following topics were included in the extraction form: bibliographic information; type of cognitive impairment, exploitation type, study design, methods, country, sample size, sample characteristics, summary of findings, and advances and limitations. Initial data extraction was completed by one reviewer. This was checked by a second reviewer, and any disagreements were moderated by the involvement of a third reviewer.

### Data Synthesis

Due to study heterogeneity related to population, study design, and outcomes reported, statistical pooling of data for meta-analysis was not possible. Instead, a narrative approach to synthesis was undertaken based on the guidance for the conduct of narrative synthesis in systematic reviews developed by Popay et al. (2006). This team-based approach involved discussion and critical reflection to develop a preliminary synthesis, explore the relationships between and within studies, and assess study robustness.

## Results

The search strategy identified a total of 6,789 results (6,671 via electronic database searches and 118 from the grey literature). After removing duplicates (222) and non-empirical studies (495), titles and abstracts were screened for potential eligibility. Following the screening, 5,671 citations were removed, with the remaining 106 full texts reviewed. A further 86 articles were excluded at the full-text stage. There were two main reasons for excluding full-text papers: not reporting empirical data and not focusing on cognitive impairment as a vulnerability to exploitation.

A total of 20 studies met the inclusion criteria (see Table 1). As per the narrative synthesis approach, the studies were initially grouped by study design and population. They were then re-grouped by type of exploitation and the most common cognitive impairments were explored. The results of the analysis will, therefore, be presented firstly by briefly considering the study design and population and, secondly by providing a more detailed thematic analysis by type of exploitation, in descending order of research quantity—that is, starting with those types of exploitation which have garnered most research attention. In analyzing and reporting each type of exploitation, the focus is on identifying what evidence (if any) exists of an association between that type of exploitation and the social or personal characteristics of the victim of exploitation. Social characteristics include confounding factors, such as unemployment, poverty, and homelessness. Personal characteristics include any type of cognitive impairment, and characteristics such as gender, ethnicity, and age. The analysis will not draw on every paper identified by the scoping review as full information is provided in Table 1. Rather, the focus is on drawing out the main themes which emerged from the evidence available.

**Table 1.** Summary of Paper Characteristics.

No.	Bibliographic Information of the Article	Cognitive Impairment	Exploitation Type	Study Design	Methods	Country	Sample Size	Sample Characteristics	Finding Summary	Advances and Limits
1	Burnett et al. (2020)	Cognitive decline Intellectual disability	Financial exploitation	Quantitative	Secondary data analysis using machine learning and penalized generalized linear modeling	United States	8,800 individuals resulting in 73, 352 senior mistreatment cases.	Community dwelling adults aged 65 years and older with at least one case of substantiated abuse between January 1, 2009 and December 31, 2014	Financial exploitation represented 29% of the cases with 78% being pure financial exploitation. Suicide ideation, the presence of drug paraphernalia, needing assistance and contentious relationships with others were the most important factors for differentiating financial exploitation versus non-financial exploitation. Hybrid financial exploitation with one other type of mistreatment present) was predicted by intellectual disability as well as the number of social factors.	The first study to report evidence linking intellectual disability with hybrid financial exploitation. The analysis is limited by the definitions of financial exploitation and investigation techniques used for the original case reports.
2	Cole (2018)	Mental health Substance misuse	Sexual exploitation	Mixed methods	Cross-sectional survey (closed and open questions) administered via telephone interview	United States	323	Service providers in agencies that supported at-risk youth	The most frequently mentioned vulnerability factors were compromised parenting/unstable home, material need, substance misuse (by the minor or their parents), developmental issues (e.g., age) and the child’s mental health and feelings about self. There were no significant differences in mentioned vulnerability factors between respondents who worked exclusively with boys or girls.	The study findings are limited as minors were not included directly and the analysis is based on professional reports.
3	Countryman-Roswurm and Bolin (2014)	Mental health Substance misuse	Domestic minor sex trafficking	Quantitative	Pre -post study conducted over a 3 month period following attendance at a psychoeducation program	United States	23	Runaway, homelessness and street youth at risk of domestic minor sex trafficking	Risk factors reported by youth: parents not married, had been in state custody, had stayed in a shelter/group home, had been pushed/shoved/grabbed in anger by a caregiver, someone caring for them had slapped them in the face or head, they had thought about harming themselves, they had used alcohol and they had used drugs.	The sample size is small. The reliability and validity of the instrument used to assess risk and resiliency experience is not known due to its early stage of development.
4	Drommi et al. (2021)	Dementia (phrased as organic diseases that impair critical faculties)	Financial exploitation	Quantitative	Secondary analysis of cases brought to the attention of judicial authority between 2010 and 2019	Italy	979 reported offenses	Reports of offenses under the Penal Code and came to the attention of the judicial authority	Financial exploitation (*n* = 240) was the second more reported form of abuse. Solitude, isolation and the presence of organic diseases were the main risk factors.	Based on reported cases which may not reflect the prevalence of issues
5	Faccini and Allely (2017)	Autism	Criminal exploitation	Qualitative	Analysis of public cases	Not all origins of case studies are identified. However, United Kingdom, United States and Norway are reported.	Seven cases	Cases reported in the public domain which are aligned with the degree of radicalization scheme.	No conclusive evidence supporting the notion that people with Autism are more violent. However, some risk factors are noted that increase vulnerabilities. Including: special interests, fantasy, obsessionality, the need for structure, communication styles, “need to matter”, social connection and disregard for other attachments.	Mental health vulnerabilities co-occur with other factors to increase risk. There is a need to investigate the order in which these co-occurring risk factors present and the pathway into terrorist acts.
6	Franchino-Olsen et al. (2020)	Intellectual disability (Low cognitive ability)	Sexual exploitation (described as sex trafficking)	Quantitative	Analysis of wave 1 and 2 from the Add Health longitudinal survey. Measures for physical disability, low cognitive ability and minor sex trafficking.	United States	5,430	Young people and their parents were recruited to wave 1 of the study in 1994/1995. Young people were aged between 12 and 18 years. Study sample was drawn from female respondents who were age 18 at wave 2.	9.7% of participants with low cognitive ability had experienced minor sex trafficking compared to 2.16% without low cognitive ability. Girls with low cognitive ability had 4.86 times greater odds of experiencing minor sex trafficking compared to girls with higher levels of cognitive abilities.	Increased likelihood of girls with low cognitive abilities being sex trafficked may be attributed to an inability to identify risky situations and more likely to be compliant with the wishes of the exploiter. Data reported is based on girls who reached adulthood 20 years ago. Questions included in the survey re sex trafficking lacked specificity.
7	Franklin and Smeaton (2017)	Intellectual disability	Sexual exploitation	Mixed methods	Online survey across all Local Authorities in the United Kingdom. Online survey of services supporting vulnerable or disabled young people. 8 Interviews with 9 statutory and 10 voluntary 11 sector stakeholders. Interviews with young people with learning disabilities.	United Kingdom	Total across all parts of study: 84	Local Authorities across England, Scotland, Wales, and Northern Ireland: Specialist organizations supporting young people: 23 (14 from child sexual exploitation specific services and from those support disadvantaged young people generally) Key stakeholders working in child sexual exploitation and/or learning disabilities: 34 (11 statutory sector and 23 voluntary). Young people (see details in article below): 27	Young people with learning disabilities are particularly vulnerable to child sexual exploitation due to: overprotection, disempowerment, social isolation, collective failure to teach sex and relationship education, inability to recognize their emerging sexuality as they age and failure of adults to notice exploitation.	While the vulnerability of disabled children to abuse is well documented, the debate re child sexual exploitation has to date neglected this increased risk. This group also face specific barriers when accessing support. More detailed studies are required to investigate both how we can prevent and respond to child sexual exploitation for young people with learning disabilities.
8	Franklin and Smeaton (2018)	Intellectual disability	Sexual exploitation	Qualitative	Interviews	United Kingdom	27	Young people aged 12–23 years. Fifteen participants had experienced sexual exploitation and 12 were deemed at risk of exploitation	Identified themes: disclosure, what worked, helping young people to understand, supporting young people to access services, working holistically with parents and carers, outcomes and young people’s recommendations for policy and practice.	Young people often did not understand that what was happening to them was abuse and their impairments were used to manipulate them. Data was only collected from young people who had been identified and were receiving some support.
9	Hestia (2020)	Mental health Substance misuse Intellectual disabilities	Criminal exploitation	Mixed methods	Analysis of initial assessments Interviews with clients, advocates, and modern slavery professionals	United Kingdom	47	1,200 initial assessments analyzed. Client interviews: 18 Advocate interviews: 26 Modern slavery professional interviews: 14	From 1,200 initial assessments, 62 cases of criminal exploitation were identified and of those 47 individuals had reported vulnerabilities. Thirty-four percent had mental health difficulties, 17% substance misuse, and 9% learning difficulties.	No recommendations or discussion regarding the role or vulnerabilities in relation to criminal exploitation.
10	Kenny et al. (2020)	Mental health Intellectual Disability	Sexual exploitation	Quantitative	Comparative analysis of confirmed cases of commercial sexual exploitation versus at-risk cases and assessed accessing support. Measures: Child: clinical diagnosis, trauma symptom checklist for children, University of California at Los Angeles Post Traumatic Stress Disoder (UCLA PTSD) Reaction Index for DSM 5 (child) and youth self-report. Parent: child behavior checklist and UCLA PTSD (parent)	United States	96 (56 confirmed cases and 40 at risk cases)	Girls aged 12–18 years and their parents	Sixty-seven percent of participants across both groups had at least one DSM-5 diagnosis. There were no significant differences across the groups based on diagnosis. A marginally significant difference was reported on the anxiety scale with the at-risk groups scoring higher. A significant difference was also found in relation to parent-related school behavior with at-risk girls more likely to be in a special education class.	Vulnerabilities in the at-risk population such as anxiety and being in a special education class indicate points for early intervention and prevention. Limitations include data being predominantly based on self-report and the use of categorical variables to code all types of abuse. Severity and extent of abuse histories need to be scrutinized in future studies.
11	Landers et al. (2017)	Mental health	Sexual exploitation	Quantitative	Florida version of the child and adolescent needs and strengths questionnaire administered at the entrance to a support program. Domains of interest were: trauma, exploitation, life domain functioning, health, youth behavioral/emotional needs, youth risk behaviors and youth strengths	United States	87	Youth aged 9–18 years admitted to a treatment program	Emotional and behavioral health symptoms: oppositional behavior (77.7%), adjustment to trauma (67.9%), depression (62.3%), conduct disorder (54.8%), anger control (54.2%) and anxiety (51.2%). Substance use that interferes with life function was noted in 46.9% of young people.	Sexually exploited youth have a greater number and more severe mental and behavioral health issues in comparison to other children who has experienced complex trauma. More in depth consideration of identified factors is needed particularly in relation to intervention.
12	Lichtenberg et al. (2020)	Cognitive decline	Financial exploitation	Quantitative	Structured questionnaires assessing financial decision making and cognition	United States	200	Community dwelling older (over 60 years) adults without diagnoses of dementia	Financial exploitation risk increases with either decisional deficiencies or cognitive decline. However, the increase is more noticeable with a combination of both decisional impairment and possible cognitive decline.	Link between financial decision-making deficits, psychosocial vulnerabilities related to financial matters and financial exploitation was supported at both the statistical significance and clinical utility level. Clinical utility is data is based on a small sample with a low base level of incapacity.
13	Lichtenberg et al. (2016)	Mental health	Financial exploitation	Quantitative	Structured questionnaires administered by telephone or left behind questionnaire as part of a longitudinal study. Data collected on: age, gender, ethnicity, years of education, marital status, depression, functional limitations, self-rated health, annual income, cognition, social needs, financial scams, and financial satisfaction.	United States	4,661	Respondents participating in the Health and Retirement Survey—a longitudinal study which started in 1992	Higher levels of depression are a significant predictor of fraud victimization. For those who had clinically significant symptoms of depression 5.7% experience fraud compared to 3.9% for the rest of the sample. For those who have clinically significant depression and also the lowest 10% in social needs fulfillment was more than twice as high compared to the rest of the sample	Those with depression are more likely to be socially isolated and lonely. They may be more vulnerable to the actions of others who are socially skilled. Two main limitations noted: the samples are not independent and participants are not included if they have a significant cognitive impairment.
14	Lichtenberg et al. (2013)	Mental health	Financial exploitation	Quantitative	Structured questionnaires administered by telephone or left behind questionnaire as part of a longitudinal study. Data collected on: age, gender, ethnicity, years of education, marital status, depression, functional limitations, self-rated health, annual income, cognition, social needs, financial scams, and financial satisfaction.	United States	4,440	Individuals who participated in the 2008 Health and Retirement leave behind questionnaire sub-study	Higher depression scores predicted an increased likelihood of experiencing fraud. High depression and low social status fulfillment (defined by the study team as the most psychologically vulnerable) was associated with a 226% increase in fraud prevalence3.	Depression and a lack of social status fulfillment have an additive effect on fraud prediction. Participants were not asked to recall the type of fraud they experienced, data collected was based on self-report and the internal consistency of the scales used were sub-optimal.
15	Manthorpe et al. (2012)	Dementia	Financial exploitation	Qualitative	Interviews	United Kingdom	15	Adult Safeguarding Coordinators	Identified themes related to the barriers and facilitators to minimizing risk for financial abuse of people with dementia	A dual focus on prevention and response is needed when supporting people with dementia who may be experiencing or at risk of financial abuse. The experiences of the respondents may have been atypical and telephone interviews can be limited.
16	Namkee et al. (2000)	Cognitive decline	Financial exploitation	Quantitative	Case controlled study. Data from intake and assessment files were reviewed from case managers notes between 1992 and 1997. All the cases had received intervention from the Protective Services for Older Adults.	United States	370	Sample divided into three mutually exclusive groups(a) self- neglecting; (b) financially exploited; (c) subject to physical, psychological or sexual abuse	The odds ratios suggest that cognitive deficit, the inability to manage one’s own finances and home ownership, had especially large effects on the likelihood of exploitation.	Risk factors for financial exploitation in older adults were identified as: cognitive deficit, the inability to manage one’s own finances and home ownership. In comparison to other groups, older adults who experience financial exploitation have fewer social supports but fewer difficulties with Activities of Daily Living (ADLs). Case files from one city in the United States may limit generalisability to other areas. The eligibility criteria for entrance into the service may have led to an overrepresentation of physically and mentally impaired participants.
17	Reid (2018)	Intellectual disabilities	Sexual exploitation	Mixed methods	Quantitative and qualitative analysis of case files comparing victims with and without intellectual disability	United States	54 (15 classified as having an intellectual disability)	Girls with evidence of initial exploitation in sex trafficking prior to 18 years	There were no statistical differences between the two groups in relation to the prevalence of early sexual abuse. The most endangering circumstance engaged in by girls with Intellectual disabilities (in comparison to control) was running away. The second most endangering circumstance was chatting on the internet. Traffickers of those with intellectual disabilities had distinct characteristics: they were more often described as taking care of the victim. In terms of social and cognitive functioning girls with intellectual disabilities were more likely to: have experienced prolonged abuse; to describe their traffickers as “boyfriends”; easily led and manipulated; and did not understand the dangerousness of the situation.	Intellectual disability compounds the effect of early sexual abuse and increases the likelihood of engaging in risky behavior. The case files were sourced from different agencies and not all contained a standardized assessment of ID. Generalisability is limited due to the focus on females in a specific geographical location.
18	Reid and Piquero (2014)	Substance misuse	Sexual exploitation	Quantitative	Analysis (structural equation modeling) of a subset of questions from the longitudinal Pathways to Desistence study.	United States	114	Subsample of child sexually exploited participants (longitudinal study *n* = 1,354 delinquent youths).	A general sequential pattern of age of onset with substance misuse and selling drugs occurring before child sexual exploitation/prostitution	The observed causal links in the earlier points of the study between drug involvement and child sexual exploitation provide supporting evidence to the psychological theories that suggest involvement in sexual exploitation is to fuel pre-existing drug use. There is a need to replicate the models with a larger sample size to ascertain generalisability. Sexual exploitation was determined by self -report
19	Samsi et al. (2014)	Dementia	Financial exploitation	Mixed methods	Cross sectional survey	United Kingdom	86	Staff working in the voluntary sector supporting older adults with dementia.	73/86 respondents indicated heightened personal vulnerability to abuse as a facet of dementia	A number of important themes were identified relating to the identification of risks of abuse, prevention of future abuse, and implications for practice and support
20	Twill et al. (2010)	Mental health Intellectual Disability	Sexual exploitation	Quantitative	Analysis of archival data collected as part of a program evaluation of a group home.	United States	22	African American girls (12–17 years) who had been charged with prostitution and received support of the group home program.	Median and Mean IQ was 70. 13 participants had IQ scores that may have qualified them for special educational services. On average each participant had at least two primary mental health conditions. Post Traumatic Stress Disorder (PTSD) was the most prevalent followed by depression.	Girls who come to the attention of the courts for prostitution have complex social and psychological histories. The small, convenience sample means that the study can only be considered descriptive and causal inferences cannot be made.

### Study Design and Population

Of the included studies, the majority (*n* = 12) used a quantitative study design, five were mixed methods and three qualitative. Sample sizes ranged from 7 to 8,800. Most of the included studies were from the United States (*n* = 13), with several from the United Kingdom (*n* = 5), one from Italy, and one which included data from multiple geographical locations. Seven studies focused on children and young people aged 25 or younger, 2 on working-age adults and 6 on older adults; 3 considered service provider perspectives. One paper included young people and their parents, and another paper included young people and service providers. Three papers focused on a female-only sample.

The types of exploitation reported included sexual (*n* = 10), financial (*n* = 8), and criminal (*n* = 2) exploitation. Intellectual disability (*n* = 8) and mental health (*n* = 8) were the most widely reported forms of cognitive impairment, but there were also a smaller number of papers which addressed substance misuse (*n* = 4), cognitive decline (*n* = 3), dementia (*n* = 3), and autism (*n* = 1). Several papers reported more than one type of cognitive impairment; for example, intellectual disability *and* cognitive decline, or mental health *and* substance misuse (see Table 1 for full details).

### Sexual Exploitation

Sexual exploitation is a contested area, with some arguing that all sex work is a form of exploitation and others that a distinction can be made between those who choose sex work and those who are coerced and exploited into sex work (Gerassi, 2015). In line with our definition of exploitation, we included papers where individuals were either forced into engaging in sex work by a third party, traded sex for goods or services (e.g., illicit drugs or alcohol), or were minors. A total of 10 papers were included in the review. All the included papers focused on sexual exploitation of children or young people with cognitive impairment. Of these, most papers were explicitly focused on girls or young women (aged 25 or younger), but some papers did not provide information about sex/gender and may have included participants of both sexes/all genders.

Within the literature on sexual exploitation, intellectual disability was the most frequently reported cognitive impairment (*n* = 4) (Franchino-Olsen et al., 2020; Franklin & Smeaton, 2017, 2018; Reid, 2018). Mental health was considered in one paper (Landers et al., 2017), and one explored substance misuse (Reid & Piquero, 2014). Two papers examined both substance misuse and mental health (Cole, 2018; Countryman-Roswurm & Bolin, 2014), while two others investigated mental health and intellectual disabilities (Kenny et al., 2020; Twill et al., 2010). Twill et al. specifically focused on the experiences of African American girls. Contextual factors associated with further increased vulnerability to sexual exploitation included living in an unstable home, being in state custody, staying in a shelter, and running away from home (Cole et al., 2016; Countryman-Roswurm & Bolin, 2014; Reid, 2018).

In comparison to other children who had experienced complex trauma, those who were sexually exploited had more mental and behavioral health issues (Landers et al., 2017; Twill et al., 2010). Twill et al. explored the experiences of African American girls who had been charged with prostitution and received support from a group home. Of the 22 participants, 13 had psychological or psychiatric records which could be used to determine whether a mental health issue was present (the other nine participants had incomplete records) (Twill et al.). On average, participants had a diagnosis of at least two primary mental health problems. Three participants were identified as having at least three Axis One mental health disorders (Twill et al.). In Landers et al. study, similar findings were noted with 77.7% of participants exhibiting traits related to oppositional behavior, 62.3% with depression, and 51.2% with anxiety. Problematic substance misuse (defined as interfering with life functioning) was found in 46.9% of participants (Landers et al. 2017).

Studies by Franchino-Olsen et al. (2020), Franklin and Smeaton (2017, 2018), and Reid (2018) identify the presence of an intellectual disability as increasing the likelihood of sexual exploitation. For example, girls with “low cognitive ability” were found to have 4.86 times greater likelihood of experiencing sex trafficking as a minor compared to participants with higher levels of cognitive ability (Franchino-Olsen et al.). Potential reasons for this increased level of vulnerability proposed by Franklin and Smeaton include overprotection, disempowerment, social isolation, a failure to teach sex and relationship education, and a failure on the part of adults to notice exploitation. Girls with intellectual disabilities were also found to be more likely to engage in activities such as running away and chatting to people on the Internet (in comparison to controls) which puts them at greater risk of exploitation (Reid, 2018). Other factors that increased vulnerability to sex trafficking were being easily led and manipulated and not understanding the gravity of the situation (Reid, 2018). Reid also suggests that exploiters of those with intellectual disabilities had specific characteristics and were more often described as “taking care of the victim”.

### Financial Exploitation

Financial exploitation refers to the use of coercion or deception to facilitate the exchange of money or assets. A total of eight studies discussed financial exploitation and, for all, the population of interest was older adults living with cognitive impairment (defined either as dementia, cognitive decline, or intellectual disability). Hybrid financial exploitation (financial exploitation with one other type of maltreatment present) was predicted by the presence of intellectual disability and other social factors (Burnett et al.). These include being unable to afford housing, food, medical care, and medications (Burnett et al., 2020). Other contextual vulnerability factors highlighted in the papers were being close to retirement, having worries about retirement, social isolation, physical health issues, being a homeowner, and lower socioeconomic status (Manthorpe et al., 2012; Namkee et al., 2000; Samsi et al., 2014).

Samsi et al. (2014) interviewed frontline practitioners who identified that dementia was a vulnerability factor for financial exploitation. For individuals experiencing cognitive decline, Lichtenberg et al. (2013, 2016) highlighted that the presence of other mental health issues such as depression and psychological vulnerabilities (e.g., susceptibility to undue influence) increased the likelihood of exploitation occurring. While not related to intellectual/cognitive ability per se, factors such as financial decision-making capacity, ability to understand financial choices, and carrying out activities of daily living were highlighted as factors that both increase the likelihood of exploitation and as potential mitigators and points for preventative intervention (Lichtenberg et al., 2020; Namkee et al., 2000).

### Criminal Exploitation

Two studies focused on criminal exploitation. Also known as forced criminality, this form of exploitation involves the use of coercion and control to force individuals to engage in illicit activities such as forced begging, pick pocketing, shoplifting, drug trafficking for another’s financial gain, or terrorism. Hestia (2020), a United Kingdom-based NGO, found that out of 47 clients who experienced criminal exploitation and reported vulnerabilities, 60% had some degree of cognitive impairment, including mental health problems (34%), misuse of drugs and/or alcohol (17%), and learning difficulties (9%). Autism was identified as a “contextual vulnerability” in cases of radicalization examined by Faccini and Allely (2017), where individuals were criminally exploited by being groomed toward undertaking acts of terrorism. No conclusive evidence was noted which suggested people with autism were more violent. However, characteristics such as having special interests, obsessionality, a need for structure and social connection, and disregarding other attachments made it easier for an individual to engage in these activities (Faccini & Allely).

## Discussion

Given the prevalence of cognitive impairment within society and increasing awareness of exploitation, only a small amount of research exploring cognitive impairment as a vulnerability to exploitation was found. The limited available evidence was partial in its coverage both of different types of exploitation and of different types of cognitive impairment (see Table 2).

**Table 2.** Critical Findings.

• Three types of exploitation were reported in the literature: sexual exploitation, financial exploitation, and criminal exploitation. • Intellectual disability and mental health were the most frequently described forms of cognitive impairment. • There is a dearth of literature exploring the relationship between forced labor and cognitive impairment and exploitative familiarity and cognitive impairment. • There are demographic gaps in the evidence base. No studies were identified which focused specifically on the sexual exploitation of men and boys. Most studies investigating financial exploitation focused on older adults. • Many studies were excluded from the review as they addressed cognitive impairments (e.g., trauma) resulting from exploitation. The presence of both causal and consequential literature related to the relationship between cognitive impairment and exploitation indicates the complexity of the dynamics and difficulties in identifying cause and effect.

### Gaps in Knowledge: Types of Exploitation

The largest, and perhaps most unexpected, gap in knowledge revealed by this scoping review was that there were no peer-reviewed studies that directly addressed the relationship between cognitive impairment and labor exploitation. The only work which considered this at all was a single research study conducted by a United Kingdom-based NGO (Hestia, 2020). This is despite the fact that labor exploitation is one of the types of exploitation most commonly identified both in the United Kingdom (Home Affairs Committee on Human Trafficking, 2023) and globally (see the International Labour Organisation’s Forced Labour Observatory (International Labor Organization, n.d.)), and despite there being clear examples of labor exploitation of people with cognitive impairment known to United Kingdom authorities and reported in the mainstream United Kingdom media (BBC News, 2019; Lincolnshire Safeguarding Adults Board, 2019). Perhaps just as surprisingly, there were also no studies identified, that explored the relationship between cognitive impairment and exploitative familiarity (“mate crime” and “cuckooing”). Although there is a small but burgeoning literature on exploitative familiarity, so far this is limited either to think pieces (e.g., Doherty, 2020; Grundy, 2011) or studies that talk in general terms about disabled people rather than explicitly considering learning disability, autism, or any other cognitive impairment (see e.g., Macdonald et al., 2022).

Of all forms of exploitation, it was sexual exploitation that had garnered the most attention from researchers. However, even within this field, it was notable that more attention has been paid to some groups than others. Importantly, all papers that addressed vulnerability to sexual abuse either had an explicit focus on girls and young women (defined as those aged 25 years or older) or failed to report the sex/gender of victims. No papers were identified that focused on the sexual exploitation of boys, young men or women aged older than 25 years with cognitive impairments. This may reflect the fact that girls and women are more vulnerable to sexual exploitation than boys and men. However, another gap, given aging populations, was that no papers addressed the sexual exploitation of older people with dementia or cognitive decline. Earlier studies have suggested that sexual abuse of older adults is something which society would prefer to pretend did not occur (Jeary, 2004).

By contrast, most papers (6/8) which explored financial exploitation of people with cognitive impairments focused on older people with either dementia or cognitive decline. This area of research interest may reflect the increasing wealth of the now-aging “baby boomer” generation (those born in the aftermath of WW2, roughly from 1946 to 1964) and their commensurate vulnerability to financial exploitation. Such exploitation may take many forms—for example, a recent paper in the British Medical Journal (Hamilton & West, 2021) highlighted the emergence of “predatory marriage”, whereby older, typically home-owning, adults with dementia or cognitive decline are targeted by exploiters who marry them in order to inherit their property and other financial assets. However, working-age adults with cognitive impairments may also be vulnerable to financial exploitation and the literature here was much scarcer. There were just two papers, both quantitative, which considered vulnerability to financial exploitation among people with mental health difficulties (Cole, 2018; Countryman-Roswurm & Bolin, 2014) and just one which considered intellectual disability (this was in addition to cognitive decline and not as the sole factor considered) (Burnett et al., 2020). This is despite the fact that financial *abuse* is known to be more prevalent among people with intellectual disabilities than among the general population (Abbott & Marriot, 2013; Fisher et al., 2016). This may reflect a disconnect within practice and research between the general concept of “abuse” and the specific concept of “exploitation.”

Only two papers examined cognitive impairment in relation to criminal exploitation, with one focusing on autism and grooming for terrorist offenses (Faccini et al., 2017) and the other considering intellectual disability, mental health, and substance misuse in relation to modern slavery (Hestia, 2020). Much therefore remains to be understood about how cognitive impairment impacts vulnerability to criminal exploitation. For example, in the United Kingdom, criminal exploitation of young people is well understood to be associated with the trade in illegal drugs (see, e.g., Robinson et al., 2019; Wroe, 2021), and this is linked to “cuckooing” (Stone, 2018). However, the relationship between this type of exploitation and cognitive impairment has yet to be properly explored.

### Gaps in Knowledge: Cognitive Impairments and Other Demographic Characteristics

There were further gaps in the literature regarding specific types of cognitive impairments, including brain injury, autism spectrum disorders and ADHD, and their relationship to exploitation, and the relationships between cognitive impairment and other demographic characteristics.

Only one paper (Twill et al., 2010) considered the experiences of exploitation among people with cognitive impairments from racially minoritized groups, but this was not a comparative study. It is not known whether or how the experiences of racially minoritized people with cognitive impairments differ from those of white people. However, through an intersectional lens (c.f., Crenshaw, 1994), it can be hypothesized that race/ethnicity may add a further layer of potential disadvantage and vulnerability.

As noted, papers on sexual exploitation focused on girls/women. Papers that considered other types of exploitation overlooked sex/gender as a factor, so it is not known, for example, whether there are sex-/gender-based vulnerabilities in relation to the financial exploitation of older people with dementia or cognitive decline. Likewise, no papers compared people from different age groups and therefore the impact of age as an additional vulnerability remains moot. Much remains to be understood about the intersections between exploitation, cognitive impairment, and ethnicity, age, or sex/gender; comparative studies that compare key characteristics are needed.

### Other Complexities of the Literature

Several studies included in this review were conducted by health and allied professions such as psychiatrists and clinical psychologists. This is noteworthy because although these professional backgrounds afford unparalleled access to invaluable clinical data, the focus of health professionals is largely on individual rather than social factors. Therefore, some papers, particularly those that adopted a quantitative approach, provided useful evidence of association and correlations between different types of exploitation (sexual, criminal, and financial) and various individual characteristics, such as sex/gender and age, but seldom afforded an analysis of wider societal factors, that is, the socioeconomic context, including contexts—such as poverty—which are believed to increase vulnerability to exploitation. By contrast, the qualitative papers tended to provide rich contextual data that exposed the impacts of poverty and other societal factors but involved small sample sizes and may not be generalizable.

It was also notable that many papers were excluded from this review because they addressed trauma/mental health difficulties that arose *as a consequence of exploitation* (particularly trauma arising from sexual exploitation and human trafficking) but did not provide evidence that trauma/mental health difficulties had been present as a pre-existing cognitive impairment that increased vulnerability to exploitation (which was the focus of this review). The presence of both causal and consequential literature on the relationship between cognitive impairment and exploitation highlights the complexity of the dynamics at play, and it is unarguably the case that cognitive impairment may be both a cause and a consequence of exploitation. For example, among the excluded papers there were studies that evidenced the negative mental health impacts of human trafficking and sexual exploitation (Abas et al., 2013; Mekeila et al., 2018), but could not provide evidence of a causal relationship between cognitive impairment and vulnerability to exploitation.

## Conclusion

Cognitive impairment does increase vulnerability to exploitation. However, there is no simple relationship between these two variables. Personal characteristics, such as race, sex/gender, and age, also appear to have an impact on an individual’s cumulative vulnerability to exploitation (French et al., 2009), but the extent of these influences is unclear. Societal factors, including unemployment, poverty, homelessness, and adverse experiences during childhood, appear to be contextual factors that contribute to individual vulnerability to exploitation (see Table 3). All of these factors, plus happenstance, will determine whether any given individual is exploited and the manner in which they are exploited.

**Table 3.** Summary of Implications for Policy and Practice.

• Further research is needed to explore the relationships between different types of cognitive impairments and forms of exploitation. A significant gap is the lack of literature exploring forced labor and cognitive impairment. • Understanding how different demographic and contextual factors affect the relationship between cognitive impairment and exploitation also requires more research. • Practitioners working with individuals with a cognitive impairment need to be able to identify warning signs related to exploitation.

This scoping review has found evidence that cognitive impairment is a factor that increases vulnerability to exploitation. However, the limited number and disparate nature of the studies mean that it is not possible to draw definitive conclusions about how or whether cognitive impairment increases vulnerability to *all* types of exploitation, or how or whether different types of cognitive impairment result in varying increases in vulnerability to *different* types of exploitation. Moreover, while many of the studies came from the United States, and their findings are valuable and may be applicable elsewhere, they are also situated in a specific context, and there is a need for further research in different geographical settings.

## Review Limitations

There are several limitations to this review. The studies have diverse methodologies and sample sizes, and we did not conduct a quality review for studies beyond the inclusion criteria of containing primary research. For reasons of access, books were excluded from the review. Due to the language capabilities of the research team, we only included studies published in English. While every effort was made to capture all relevant papers within this review, some admissible studies may not have been caught by the search terms. While we used a range of alternative terms that could be used to describe various forms of exploitation, these were not exhaustive. Therefore, it is possible that the review may have missed studies where the phenomenon being studied was not described as exploitation but would have met our definition of exploitation.
